# An investigation into genetic contribution to the relationship between insulin resistance and birth weight

**DOI:** 10.4103/0973-3930.50709

**Published:** 2009

**Authors:** Samsad Jahan, Rahelee Zinnat, Zahid Hasan, Chowdhury Meshkat Ahmed, Samira Humaria Habib, Soma Saha, Liaquat Ali

**Affiliations:** Department of Gynecology and Obstetrics, BIRDEM, Dhaka, Bangladesh; 1 Department of Cell and Molecular Biology, BIRDEM, Dhaka, Bangladesh; 2 Department of Cardiology, BSMMU, Dhaka, Bangladesh; 3 Department of Health Economics Unit, DAB, Dhaka, Bangladesh

**Keywords:** Birth weight, genetic contribution, insulin resistance, type 2 diabetes mellitus

## Abstract

**BACKGROUND:**

Insulin resistance has been proposed to be the most likely phenotypic trait that could represent a genetic link between low birth weight and type 2 diabetes, especially in Southeast Asia. Insulin resistance can persist for many years, even decades, before the manifestation of overt diabetes. There have been many studies suggesting a strong genetic basis in the etiology of type 2 diabetes mellitus. There is also ample evidence providing a link with low birth weight and type 2 diabetes in later life. Hence, parental insulin sensitivity could well serve as a representation of the offspring's future insulin resistance state. Association between maternal insulin sensitivity and the incidence of type 2 diabetes mellitus in low birth weight babies is confounded by many factors and hence, has limited value in the determination of any genetic origin of the disease. Therefore, the present study was done to investigate the relationship between paternal insulin sensitivity and the growth parameters of the foetus to determine a genetic link between poor early growth and the increased risk of type 2 diabetes mellitus in later life.

**MATERIALS AND METHODS:**

The study was performed on 30 healthy fathers and their babies born from nondiabetic mothers. Each father underwent a low-dose short insulin tolerance test (ITT) as a measure of insulin sensitivity. Placental weight was recorded and a blood sample was collected from the placental side of the umbilical cord at birth for measurement of insulin. Measurement of birth weight, length, and head circumference were recorded and ponderal index was calculated from the formula: weight (kg)/ length (cm)^3^. Individual parameters of insulin resistance syndrome were measured in the fathers.

**RESULTS:**

The degree of insulin sensitivity, K_m_ (constant for insulin tolerance test) did not correlate with the fetal growth parameters (Ponderal Index r = 0.031, *P* = 0.870; weight of baby r = 0.010, *P* = 0.959; length of baby r = 0.087, *P* = 0.464; head circumference r = 0.280, *P* = 0.142) or with the fathers' anthropometric measures: body mass index (BMI), blood pressure, fasting glucose, insulin, and lipid profiles.

**CONCLUSION:**

The data suggest that the mechanism linking insulin resistance with low birth weight is not a genetically determined defect.

## Introduction

In this prospective study, we aimed to test the hypothesis that the mechanism linking insulin resistance in adult life with low birth weight is a genetically determined disease. There have been epidemiological studies,[1–4] family studies,[5–8] and twin studies[9–13] suggesting a strong genetic basis in the etiology of type 2 diabetes mellitus. There is also ample evidence providing a link with low birth weight and type 2 diabetes in later life.[14–17] Insulin resistance has been proposed to be the most likely phenotypic trait that could represent a genetic link between low birth weight and type 2 diabetes, especially in Southeast Asia.[4,18]

Insulin resistance persists for many decades before the manifestation of overt diabetes,[18] so that parental insulin sensitivity could well serve as a representation of insulin resistance state. Association between maternal insulin sensitivity and the incidence of type 2 diabetes mellitus in low birth weight babies is confounded by many factors and hence, has limited value in the determination of any genetic origin of the disease. Thus, this study aimed to examine the relationship between paternal insulin sensitivity and parameters of early growth in the offspring as a means of exploring the genetic mechanism linking insulin resistance with low birth weight.

## Materials and Methods

The present prospective study was carried out in the Department of Obstetrics and Gynecology, Bangabandhu Sheikh Mujib Medical University (BSMMU) and the Bangladesh Institute of Research and Rehabilitation in Diabetic, Endocrine and Metabolic disorders (BIRDEM) and in the Research Division, BIRDEM, Dhaka, from April 1998 to March 1999.

The study was performed on 30 healthy fathers of babies born to nondiabetic women delivering consecutively at the Department of Obstetrics and Gynecology in the BSMMU and BIRDEM hospitals. The study also included these 30 babies and their mothers. Inclusion criteria: healthy fathers of the babies born to nondiabetic women delivering consecutively in the BSMMU and BIRDEM hospitals. Exclusion criteria: i) multiple pregnancies of the mothers who smoked >10 cigarettes per day; ii) any parent suffering from medical or endocrine diseases (including diabetes, hypertension, epilepsy, or ischemic heart disease in fathers); iii) mothers who reported late for antenatal check-up; iv) mothers whose antenatal check-up attendance was poor and who suffered from hypertension, preeclampsia, or eclampsia; v) difficult deliveries, *e.g*., significant meconium or fetal distress, premature delivery (< 37 weeks) detected by clinical criteria or by ultrasound scans.

Written informed consent was obtained from the parents (subjects). The fathers' details including age, alcohol history, drug history, social class, and history of diabetes or other diseases were recorded. The mothers' age, smoking and alcohol history, history of last menstrual period, history of diabetes or other diseases, drug history, previous obstetric history, parity, height, weight, and body mass index were recorded. The babies' gender and birth order were recorded.

## Investigations

Fathers: Each father underwent a low-dose short insulin tolerance test (ITT), a modification of Bonora's method,[19] as a measure of insulin sensitivity. The fathers were invited one week prior to the low-dose short insulin tolerance test for an oral glucose tolerance test as a formal assessment of their glucose tolerance. Insulin levels were measured using the ELISA method; glucose was measured using the glucose oxidase colorimetric method, and lipoprotein was measured by using an enzymatic method.

Mothers: A blood sample was collected for future DNA analysis. Placental weight was recorded and a blood sample was collected from the umbilical cord at birth for insulin measurement and DNA analysis.

Babies: Measurements of birth weight, length, and head circumference were recorded and ponderal index was calculated from weight (kg)/length (cm^3^). Statistical analysis was done by using SPSS (Statistical Package for Social Sciences) software. Standard deviation was taken as measure of variation and the frequencies of the data were expressed as mean ± SD. The relationships between paternal insulin sensitivity calculated from ITT and ponderal index, birth weight, birth length, and head circumference have been explored using a correlation coefficient test. These data were analyzed by using multiple linear regressions to examine the dependence of ponderal index on other variables. Maternal body mass indices and gestation periods were divided into tertiles to evaluate their relationship with ponderal index. The ANOVA test was performed to determine the differences between the groups. *P* < 0.05 was taken as the minimum level of statistical significance.

## Results

### Mothers

The age of the mothers ranged from 18 to 35 years (mean ± SD = 25.00 ± 5.98 years). The body mass index (BMI) of the mothers had a mean ± SD of 24.2 ± 2.37 kg /m^2^ (15.43–27.34 kg/m^2^). The percent BMI range was 74.91–133.9 with a mean ± SD of 117.39 ± 11.7. The height of the mothers ranged from 1.52 to 1.80 meters and the mean ± SD was 1.58 ± 0.12 meters. The weight of the mothers ranged from 52 to 70 kg with a mean ± SD of 60.3 ± 5.57 kg.

Twenty-eight (93%) mothers were from urban areas and two (70 %) were from rural areas; this ratio was 14:1. The mean blood pressure of the mothers ranged from 70 to 103 mm Hg with a mean ± SD of 92.68 ± 8.09 mm Hg. The gestational age ranged from 37.5 to 41.0 weeks with a mean ± SD of 38.96 ± 1.16 weeks. The parity of the mothers ranged from 0 to 5. The weight of the placenta varied from 400 to 600 g with a mean ± SD of 490 ± 40 g.

The social, anthropometric, clinical, and obstetrical data characteristics of the mothers have been shown in Table 1.

**Table 1:** Anthropometric, clinical, and obstetrical characteristics of the mothers

Characteristics (*n* = 30)	Range	Mean ± SD
Age (years)	18–35	25.00 ± 5.36
Height of Mothers (m)	1.52–1.80	1.58 ± 0.12
BMI (kg/m^2^)	15.43–27.43	24.2 ± 2.37
Percent BMI	74.91–132.70	117.39 ± 11.7
Systolic BP (mm Hg)	90–140	120.00 ± 12.65
Diastolic BP (mm Hg)	60–85	79.03 ± 6.64
Mean BP (mm Hg)	70–103.33	92.68 ± 8.09
Gestational age (weeks)	37.5–41.0	38.96 ± 1.16

### Babies

Out of 30 babies, there were 21 male and nine female babies, the male-female ratio being 7:3. The weight of the babies ranged from 2.1 to 3.5 kg with a mean ± SD of 2.66 ± 0.41 kg. The length (crown to heel length) of the babies ranged from 47 to 50 cm with a mean ± SD of 48.4 ± 1.26 cm. The mean ± SD of the ponderal index of the babies was 23.27 ± 2.86 kg/m^3^ and the range was 18.08–28.90 kg /m^3^. The head circumference ranged from 32 to 36 cm with a mean ± SD of 34.13 ± 1.24 cm. The serum cord insulin level of the babies ranged from 2.12 to 29.76 mIU/L with a mean ± SD of 9.18 ± 5.85 mIU/L.The anthropometric data, characteristics of the babies, and their cord insulin concentrations have been shown in Table 2.

**Table 2:** Anthropometric characteristics suggesting fetal growth

Characteristics (*n* = 30)	Range	Mean ± SD
Weight (kg)	2.1–3.5	2.66 ± 0.41
Length (cm)	47–50	48.4 ± 1.26
Ponderal index (kg /m^3^)	18.08–28.90	23.27 ± 2.86
Head circumference (cm)	32–36	34.13 ± 1.24
Cord insulin of fetus (mIU/L)	2.12–29.76	9.18 ± 5.85

### Fathers

The mean ± SD age of the fathers was 33.55 ± 5.98 years (range: 26–50 years), mean ± SD height was 1.63 ± 8.3 meters (range: 1.47 to 1.77 m). The BMI varied from 15.32 to 35.78 kg/m^2^, (mean ± SD = 22.83 ± 4.09 kg/m^2^) and the mean ± SD percent BMI was 103.49 ± 18.56 (range: 69.33–161.90). The weight of the fathers ranged from 45 to 98 kg (mean ± SD = 60.41 ± 11.36 kg). The mean blood pressure of the fathers ranged from 70 to 103.33 mm Hg (mean ± SD = 83.82 ± 9.28 mm Hg).

The anthropometric characteristics of the fathers reflecting the body adiposity distribution as a measure of insulin resistance showed: mid-arm circumference range = 21–38 cm, subscapular to triceps skin fold thickness ratio mean ± SD = 1.26 ± 0.34 (range: 0.73–1.88); waist-hip ratio mean = 0.95 (range: 0.65–1.04). The half-life for glucose derived from the insulin tolerance test varied from 4.63 to 13.76 minutes. The KITT for the low-dose, short- term insulin tolerance test varied from 0.0509 to 0.1497. The clinical, anthropometric, and insulin sensitivity characteristics of the fathers have been shown in Table 3.

The lipoprotein profile of the fathers revealed that the total cholesterol range was 109.96–307.00 g/dL (mean ± SD = 162.16 ± 38.92 mg/dL); triglyceride range was 41.47–171.23 g/dL (mean ± SD = 95.60 ± 28.37 g/dL); LDL cholesterol range was 70.71–242.74 mg/dL (mean ± SD = 114.69 ± 34.20 mg/dL); HDL cholesterol range was 15.97–48.88 mg/dL (mean ± SD = 28.72 ± 6.69 mg/dL); and the fasting serum insulin range was 1.60–28.24 mIU/L (mean ± SD = 9.83 ± 5.34 mIU/L). The metabolic characteristics of the fathers have been shown in Table 4.

Significant correlations were observed between indices of fetal growth and factors such as the gender of the babies as well as maternal factors such as gestational age and maternal basal metabolic rate. Parity of the mothers was weakly related to the fetal growth parameters. The strongest correlation was found between gestational age and the ponderal index (r = 0.474, *P* = 0.008). There was also a good correlation between gestational age and weight of the baby and ponderal index (r = 0.479; *P* = 0.007 and r = 0.474; *P* = 0.008 respectively).

There was correlation between the body mass index of the mother and the fetal ponderal index (r = 0.379, *P* = 0.039). Male gender of the babies correlated well with the head circumference of the babies (r = 0.448, *P* = 0.015).

Parity was also correlated with ponderal index of the fetuses but this correlation was weak (r = 0.320, *P* = 0.084). Weaker correlation was found between maternal age and ponderal index. The maternal age related inversely with ponderal index (r = -0.235, *P* = 0.204). Table 5 shows the correlations between maternal factors and the growth parameters of the fetuses (Pearson's correlation) along with their significance (*P* value).

Similar increasing trends were seen from groups 1 to 3 for the means of the ponderal index, mean birth weights, and maternal BMI values. The means of the ponderal index were 21.88 kg/m^3^, 22.53 kg/m^3^, and 25.59 kg/m^3^ for groups 1, 2, and 3 respectively. The difference between the groups was significant (F = 7.218, *P* = 0.003).

In a similar way, when maternal BMI values were divided into three groups, the mean birth weights and ponderal index of the babies increased as each group showed increased BMI (group 1 BMI up to 24 kg/m^2^, group 2 BMI 24.1–25.9 kg/m^2^, and group 3 BMI was ≥ 26 kg/m^2^). The mean ponderal index was 20.93 kg/m^3^ in group 1, 23.12 kg/m^3^ in group 2, and was 24.80 kg/m^3^ in group 3. There was a significant difference between the groups (F = 3.943, *P* = 0.032).

The mean values of the babies' weight were 2.46 kg in group 1, 2.636 kg in group 2, and 28.00 kg in group 3. Here, the difference was not significant between the groups (*P* = 0.314). The other parameters of growth such as length and head circumference of the babies also did not show any significant relationship with maternal BMI (*P* = 0.423 and *P* = 0.432 respectively). The distribution of the means of the ponderal index, birth weight, length, and head circumference of the babies has been shown in relation to the maternal BMI values in Table 6.

**Figure 1:**
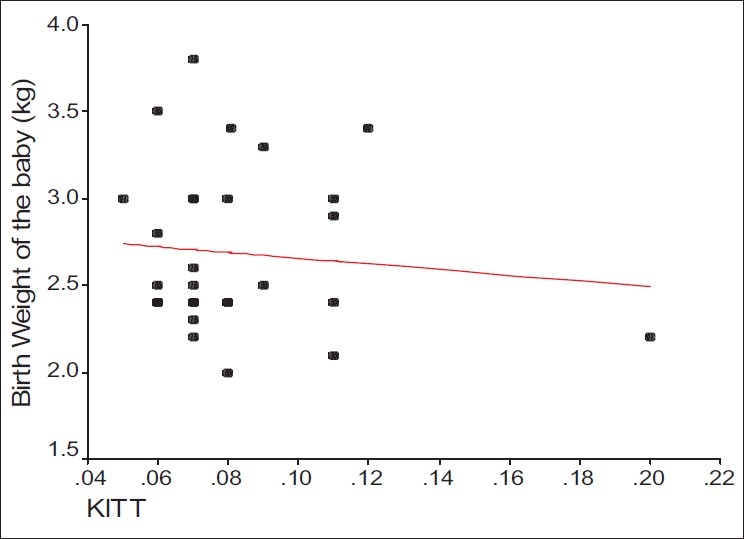
Correlation between KITT and weight of the babies

**Figure 2:**
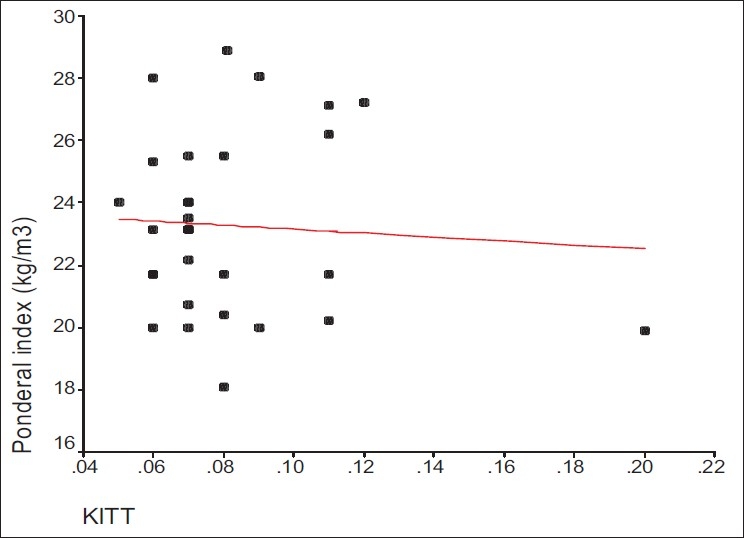
Correlation between KITT and ponderal index of the babies

We applied multiple regressions to analyze the simultaneous relationships of maternal body mass index, gestational age, parity, and insulin resistance with the ponderal index. The effect of gestational age on ponderal index remained statistically significant (*P* = 0.0124) as did the effect of maternal body mass index (*P* = 0.0044). Neither degree of insulin sensitivity nor other clinical parameters of insulin sensitivity of the father correlated with the fetal growth parameters [Figures 1, 2].

The correlations of the fathers' factors suggesting insulin sensitivity and other factors with the fetal growth parameters have been shown in Table 7 with their significance (r values and *P* values).

**Table 3:** Anthropometric, clinical, and insulin sensitivity characteristics of the fathers

Characteristics (*n* = 30)	Range	Mean ± SD
Age (years)	26–50	33.55 ± 5.98
BMI (kg/m^2^)	15.32–35.78	22.83 ± 4.09
Percent BMI	69.33–161.90	103.49 ± 18.56
Mean BP (mm Hg)	70.00–103.33	83.82 ± 9.28
subscapular to triceps	0.73–1.88	1.26 ± 0.34
skin fold thickness ratio	0.85–1.04	0.95
waist-hip ratio	0.0504–0.1497	
Constant for ITT		

**Table 4:** Metabolic characteristics of the fathers

Characteristics (*n* = 30)	Range	Mean ± SD
Total cholesterol (mg/dL)	109.96–307.00	162.16 ± 38.92
Triglyceride (mg/dL)	41.47–171.23	95.60 ± 28.37
HDL cholesterol (mg/dL)	15.97–48.88	28.72 ± 6.69
LDL cholesterol (mg/dL)	70.71–242.78	114.69 ± 34.20
Plasma insulin (F)(mIU/L)	1.60–28.24	9.83 ± 5.34

**Table 5:** Correlations between maternal factors and the growth parameters of the fetuses

Parameters (*n* = 30)	BMI	Height	Parity	Gestational age	Gender of baby
Ponderal index	r = 0.379	r = 0.183	r = 0.320	r = 0.474	r = 0.226
	*P* = 0.039*	*P* = 0.334	*P* = 0.084	*P* = 0.008**	*P* = 0.230
Weight					
r = 0.179	r = 0.181	r = 0.239	r = 0.479	r = 0.169	
	*P* = 0.344	*P* = 0.339	*P* = 0.202	*P* = 0.007**	*P* = 0.371
Length	r = 0.298	r = 0.043	r = 0.045	r = 0.211	r = 0.000
	*P* = 0.110	*P* = 0.812	*P* = 0.812	*P* = 0.264	*P* = 1.000
Head	r = 0.177	r = 0.011	r = 0.006	r = 0.144	r = 0.169
circumference	*P* = 0.357	*P* = 0.953	*P* = 0.974	*P* = 0.440	*P* = 0.015*

* Significant relationship at the level of 0.05

** Significant relationship at the level of 0.01

**Table 6:** Relationship of ponderal index, birth weight, length, and head circumference of the babies with maternal BMI

Parameters	Maternal BMI (kg/m^2^)	F value	*P* value
Ponderal index kg/m^3^			
20.93	Up to 24	3.943	0.032
23.12	24.1–25.9		
24.80	> 26		
Weight of baby (kg)			
2.467	Up to 24	1.212	0.314
2.636	24.1–25.9		
2.800	>26		
Length of baby (cm)			
49.00	Up to 24	0.890	0.423
48.42	24.1–25.9		
48.22	> 26		
Head circumference (cm)			
34.17	Up to 24	0.867	0.432
34.38	24.1–25.9		
33.67	> 26		

**Table 7:** Correlations of the different paternal factors suggestive of insulin sensitivity and the growth parameters of the fetuses

Parameters (*n* = 30)	KITT	BMI	Mean BP	MAC	WHP	STR	Sub T
Ponderal	r =0.031	r=0.065	r=0.276	r=0.236	r=-0.218	r=0.131	r=0.093
index	*P*=0.870	*P*=0.732	*P*=0.618	*P*=0.204	*P*=0.248	*P*=0.489	*P*=0.625
Weight of baby	r=0.010	r=0.120	r=0.241	r=0.085	r=-0.146	r=0.004	r=0.148
Length of baby	*P*=0.959	*P*=0.527	*P*=0.681	*P*=0.656	*P*=0.441	*P*=0.982	*P*=0.436
Head	r=0.087	r=0.130	r=0.026	r=0.269	r=-0.868	r=0.254	r=0.151
circumference	*P*=0.464	*P*=0.492	*P*=0.822	*P*=0.151	*P*=0.721	*P*=0.176	*P*=0.426
	r=0.280	r=0.253	r=0.126	r=0.152	r=-0.095	r=0.056	r=0.285
	*P*=0.142	*P*=0.185	*P*=0.185	*P*=0.432	*P*=0.625	*P*=0.775	*P*=0.134

KITT: Constant for insulin tolerance test, BMI: Body Mass Index, MAC: Mid Arm Circumference, WHP: Waist to Hip Ratio, STR: Subscapularto-to-Tricep Ratio, Sub T: Subcutaneous Thickness

## Discussion

If type 2 diabetes mellitus is genetic in origin and disproportionate fetal growth is linked to type 2 diabetes in the future life of the fetus, then the parental diabetes status must have some link to the parameters of fetal growth. To evaluate this issue, we linked the pathophysiological markers of type 2 diabetes with the parameters of fetal growth. Although the etiology of type 2 diabetes is not exactly known, both insulin resistance and deficient insulin secretion are implicated in its pathogenesis.[20] With respect to pathogenesis of type 2 diabetes mellitus, one has to be very aware that phenotypic characterization of type 2 diabetes mellitus is not homogenous. Vadheim and Rotter[21] suggested that clinical differences in the diabetic syndromes between ethnic groups and marked differences between normal plasma glucose and insulin concentrations between different populations indicate the heterogenicity of type 2 diabetes mellitus. The authors recommended that the population-based studies of the distribution of phenotypic traits could be helpful to evaluate if the trait is controlled by a major gene or by multiple factors. A recent physiological study in Asian Indians with type 2 diabetes mellitus has suggested that Indian diabetic individuals are more insulin resistant than Caucasians.[21] The high incidence of coronary heart disease among Asian Indians in different parts of the world is consistent with this finding. Tan and his colleagues[4] have shown that Asian Indians in Singapore also have a high incidence of coronary heart disease in comparison to two other major ethnic groups. The clustering of diabetes, insulin resistance, central obesity, and low HDL in the Singaporean Asian Indians strongly suggested that insulin resistance could have been a major contributory factor towards the development of coronary heart disease. Mohan *et al*.[22] had earlier demonstrated higher insulin levels in normal and type 2 diabetic Asian Indians than in normal and type 2 diabetic Europeans. For this reason, we chose insulin resistance as the physiological phenotypic trait to evaluate the genetic link between type 2 diabetes and the fetal growth parameters. Several studies have shown that insulin resistance precedes the onset of clinical type 2 diabetes mellitus by several decades.[23] Thus, if the genetic predisposition of insulin resistance is linked to poor early growth, then parental insulin sensitivity should also be related to parameters of fetal growth. But a study of such an association between maternal insulin sensitivity and infant birth weight would be of limited value as it would be difficult to distinguish if low birth weight results from the inheritance of an insulin-resistant gene or due to poor fetal nutrition arising from an unfavourable intrauterine environment associated with maternal insulin resistance. Moreover, Stanley and her colleagues showed a progressive increase in insulin resistance in all women as pregnancy progressed and suggested that insulin resistance is physiological and likely to be mediated by pregnancy hormones operating in the fetal interest. Cortisol, progesterone, prolactin, or human placental lactogen have been suggested to be responsible for such insulin resistance during pregnancy. To overcome this problem, we aimed to explore the relationship between paternal insulin sensitivity and birth weight of the offsprings. This enabled us to examine the genetic contribution to insulin sensitivity without its confounding influence on the uterine environment. There was no evidence suggesting that insulin resistance is specifically transmitted through the maternal line so our study has provided a simple and straightforward test of the genetic hypothesis. In addition to measurement of whole body insulin sensitivity in the father, individual parameters of the insulin resistance syndrome (and the insulin response to glucose) were assessed. In this study, we could not find any trends towards the relationship between paternal insulin sensitivity and the parameters of early growth in the offsprings. Similarly, our study also failed to detect any trends towards the correlation between any individual parameters of the insulin resistance syndrome with the parameters of fetal growth. Circulating fetal insulin level also had no correlation with fetal growth parameters. Elevated concentrations could be expected from insulin-resistant infants who could also have growth impairment. The findings of our study suggest that the poor early growth and the associated increased risk of type 2 diabetes in later life are not likely to be genetic in origin, at least, if the operating mechanism dominating the pathogenesis is insulin resistance. Our findings indirectly imply that the risk of later development of type 2 diabetes could be due to environmental factors such as the intrauterine environment. In this study, we have proposed that the mechanism linking insulin resistance with low birth weight is not genetically determined. But the probability that a genetic link between low birth weight and type 2 diabetes in later life can exist through a genetic defect of the beta cells cannot be excluded. Our negative findings thus could have a sizable impact on the “thrifty phenotype” hypothesis. As the critics for thrifty phenotype hypothesis, Paneth and Susser[23] mentioned in 1995 that what was missing in the thrifty phenotype hypothesis so far was the rigorous testing by rejection and exclusion using deliberate attempts at refutation. We have rejected and excluded the most important possibility that the link between low birth weight and type 2 diabetes in later life is genetic in origin. Refutation of our hypothesis has implied that the risk of later type 2 diabetes is due to environmental factors such as the intrauterine environment. This puts Barker's hypothesis (thrifty phenotype) one step forward.

## Conclusion

Our study revealed that there is no correlation between paternal insulin sensitivity or any other clinical facet of insulin resistance with parameters of fetal growth. As our hypothesis is refuted, it implies that the risk of later development of type 2 diabetes in a growth-retarded baby is due to environmental factors such as the intrauterine environment. The findings of our results suggest that improvement in nutritional status during pregnancy might have mitigated many sufferings of the offsprings in later life.
